# An Assessment of Melatonin’s Therapeutic Value in the Hypoxic-Ischemic Encephalopathy of the Newborn

**DOI:** 10.3389/fnsyn.2019.00034

**Published:** 2019-12-10

**Authors:** Daniel P. Cardinali

**Affiliations:** Faculty of Medical Sciences, Pontificia Universidad Católica Argentina, Buenos Aires, Argentina

**Keywords:** hypoxic-ischemic encephalopathy, hypothermia, melatonin, oxidative stress, inflammation, neurodegeneration

## Abstract

Hypoxic-ischemic encephalopathy (HIE) is one of the most frequent causes of brain injury in the newborn. From a pathophysiological standpoint, a complex process takes place at the cellular and tissue level during the development of newborn brain damage in the absence of oxygen. Initially, the lesion is triggered by a deficit in the supply of oxygen to cells and tissues, causing a primary energy insufficiency. Subsequently, high energy phosphate levels recover transiently (the latent phase) that is followed by a secondary phase, in which many of the pathophysiological mechanisms involved in the development of neonatal brain damage ensue (i.e., excitotoxicity, massive influx of Ca^2+^, oxidative and nitrosative stress, inflammation). This leads to cell death by necrosis or apoptosis. Eventually, a tertiary phase occurs, characterized by the persistence of brain damage for months and even years after the HI insult. Hypothermia is the only therapeutic strategy against HIE that has been incorporated into neonatal intensive care units with limited success. Thus, there is an urgent need for agents with the capacity to curtail acute and chronic damage in HIE. Melatonin, a molecule of unusual phylogenetic conservation present in all known aerobic organisms, has a potential role as a neuroprotective agent both acutely and chronically in HIE. Melatonin displays a remarkable antioxidant and anti-inflammatory activity and is capable to cross the blood-brain barrier readily. Moreover, in many animal models of brain degeneration, melatonin was effective to impair chronic mechanisms of neuronal death. In animal models, and in a limited number of clinical studies, melatonin increased the level of protection developed by hypothermia in newborn asphyxia. This review article summarizes briefly the available therapeutic strategies in HIE and assesses the role of melatonin as a potentially relevant therapeutic tool to cover the hypoxia-ischemia phase and the secondary and tertiary phases following a hypoxic-ischemic insult.

## Introduction

According to the World Health Organization, for every day in 2015, 16,000 children aged under 5 died (World Health Organization [WHO], 2015). Forty-five per cent of those deaths were in newborns, mainly due to intrapartum-related complications and prematurity. In particular, hypoxic-ischemic encephalopathy (HIE) remains as a significant problem particularly in low-resource countries, where the rate of asphyxia is about 10-fold higher (10–20 per 1000 live births) than in developed countries (Lawn et al., 2005).

Intrapartum hypoxic-ischemic (HI) events including placental abruption, umbilical cord prolapse, obstructed labor, uterine rupture, and fetal infection result in impaired oxygenation and perfusion of vital organs in the fetus and newborn infant (Yildiz et al., 2017). HI injury to peripheral organs is often reversible (Yildiz et al., 2017). In contrast, HIE injury may lead to permanent neurological impairment. Survivors with long-term disability commonly have cerebral palsy including spastic quadriplegic and dyskinetic types (Schreglmann et al., 2019).

Knowledge of HIE pathophysiology derived in the description of potential therapeutic targets to reduce cerebral damage after asphyxia, with the consequent postulation of therapeutic strategies. This review article summarizes briefly the available therapeutic strategies in HIE and assesses the role of melatonin, a potentially relevant therapeutic tool to cover the hypoxia-ischemia phase and the secondary and tertiary phases following a HI insult. Medical literature was identified by searching databases including (MEDLINE, EMBASE), bibliographies from published literature and clinical trial registries/databases. Searches were last updated on September 1, 2019.

## Pathophysiology and Therapeutic Strategies in HIE

A complex process takes place at the cellular and tissue level due to lack of oxygen (Yildiz et al., 2017).

**FIGURE 1:**
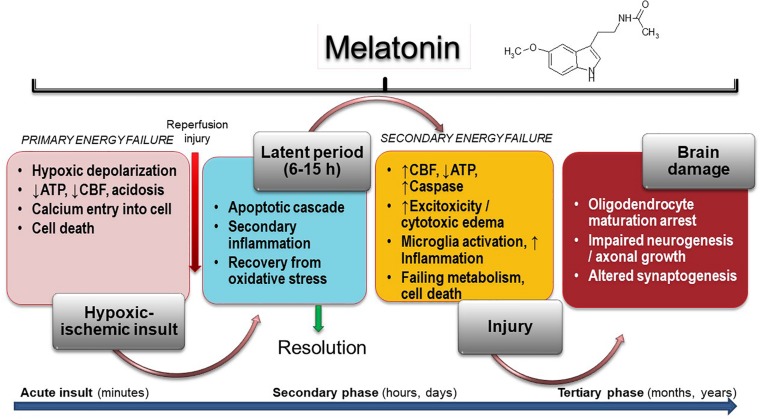
Melatonin activity in HIE. Insult results in primary (acute phase) and secondary energy failure (secondary phase) in the brain while brain damage (tertiary phase) continues to occur months to years after the injury with decreased plasticity and reduced number of neurons. Melatonin has the unique property to cover all phases including attenuation of tertiary brain damage, hence expanding the therapeutic window to long-term outcome. CFB, cerebral blood flow.

The harmful mechanisms during the development of newborn brain damage by hypoxia can be grouped based on the time elapsed since their appearance (Yildiz et al., 2017). They comprise four phases (Figure 1): (a) hypoxia-ischemia phase; (b) latent phase; (c) secondary phase; (d) tertiary phase. At first, the injury is triggered by a deficit in the supply of oxygen to cells, causing a primary energy insufficiency. Subsequently, high energy phosphate levels may transiently recover (the latent phase), this phase elapsing for 3–15 h. In the secondary phase that followed, many of the pathophysiological mechanisms of neonatal brain damage are triggered. Excitotoxicity, massive influx of Ca^2+^, oxidative stress, inflammatory reaction and eventually cell death by apoptosis or necrosis are characteristics of the secondary phase that elapses from some hours to days. Finally, a tertiary phase ensues, characterized by persistent cerebral damage for months and years after the HI injury (Yildiz et al., 2017).

Insufficient oxygen delivery to the CNS is the initial event in HIE. It is produced either by hypoxia or by impaired cerebral blood flow due to hypovolemia or impaired circulation. Commonly, a combination of both events takes place (Yildiz et al., 2017). A decrease production of ATP and lactic acidosis occur in the primary energy failure after injury (Torres-Cuevas et al., 2019). Disruption of neuronal cell membrane leads to massive calcium entry to the cell, an event mediated by activation of *N*-methyl-D-aspartate (NMDA) receptor by excitotoxic neurotransmitters (glutamate, aspartate) (Pregnolato et al., 2019). Only very few therapeutic strategies have the capacity to control the primary energy failure. As discussed below, melatonin may be one of them.

A period of latency that lasts for some hours after primary energy failure gives the possibility for interventions to curtail prospective brain damage. If they are not provided, a secondary energy failure leads to HIE (Yildiz et al., 2017). Excitotoxicity, oxidative stress, inflammation and cell death characterize the secondary energy failure that ensues 6–48 h after hypoxic/ischemia (HI). In some infants, a tertiary phase of brain damage arises due to the presence of active mechanisms preventing regeneration of neurons. These mechanisms include a low-degree inflammation, impairment of oligodendrocyte maturation, impaired neurogenesis and axonal growth, and disrupted synaptogenesis. Myelin deficits and reduced plasticity can persist for months to years after the initial injury (Fleiss and Gressens, 2012).

The ischemia-reperfusion phenomenon that occurs after perinatal asphyxia triggers the increase in reactive oxygen species (ROS), a subsequent lipid and protein peroxidation and the stimulation of apoptotic or necrotic pathways in brain cells (Castillo-Melendez et al., 2004; Miller et al., 2012; Aridas et al., 2016; Martinez-Biarge et al., 2019). The increase in ROS is detectable very early after asphyxia (i.e., up to 30 min) and may elapse for several days (Yan et al., 2005). Poorly developed innate antioxidative defense systems turn the newborn CNS very prone to oxidative stress (du Plessis and Volpe, 2002; Castillo-Melendez et al., 2013).

Neuroinflammation and oxidative damage interact each other. ROS trigger pro-inflammatory cytokine release and microglial activation, and conversely, microglia release free radicals and pro-inflammatory cytokines (Miller et al., 2012). The control of these early mechanisms of brain injury is generally considered an avenue for any promising neuroprotective strategy (Hassell et al., 2015).

The use of hypothermia as a neuroprotective therapy in neonatal HIE derives from the discovery of endogenous cooling mechanisms that are triggered by brain damage at birth (Burnard and Cross, 1958). The neuroprotective effect of hypothermia is related mainly to the reduction of brain metabolism (a 5% decrease for each degree of temperature), a situation the moderates several metabolic routes triggered after suffocation (Laptook et al., 1995).

Only a modest improvement in mortality and long-term neurological morbidity is provided by hypothermia therapy in HIE (Roka and Azzopardi, 2010; Jacobs et al., 2013; Alonso-Alconada et al., 2015). In addition, there are data indicating that hypothermia in low-income countries does not reduce mortality but rather it may aggravate prognosis in the presence of sepsis (Pauliah et al., 2013). Therefore, there is an urgent need to identify therapeutic agents effective to treat HIE.

Recent research focuses in the development of therapies that can be used in combination with hypothermia thus fostering synergy between therapeutic strategies (Hassell et al., 2015). They include erythropoietin (EPO), allopurinol, stem cells, noble gases, and melatonin.

Erythropoietin is a cytokine that is synthesized during the fetal period by the liver and postnatally by the kidney and the brain, and that acts as a growth factor and neuroprotective agent (Juul and Pet, 2015). The beneficial effect of EPO in HIE is based on its action on specific receptors present in neurons and glia, capable of developing a powerful antiapoptotic activity (favoring gene transcription of antiapoptotic, anti-inflammatory and antioxidant components) (Juul and Pet, 2015). Moreover, EPO promotes long term reparative phenomena like neurogenesis, oligodendrogenesis and angiogenesis (Jantzie et al., 2015). Up today, three phase III clinical trials are in progress on a total of 840 infants to assess the safety and efficacy of high doses of EPO (1,000 U/kg) in combination with hypothermia (Erythropoietin in Management of Neonatal Hypoxic Ischemic Encephalopathy, NCT03163589; High-dose Erythropoietin for Asphyxia and Encephalopathy, NCT02811263; Erythropoietin for Hypoxic Ischaemic Encephalopathy in Newborns, NCT03079167).

The inhibitory effect on xanthine oxidase, an enzyme involved in oxidative damage, is the base for the use of allopurinol as a therapy against HIE. In addition, allopurinol has the capacity to chelate free iron and acts as a scavenger of hydroxyl radicals (Ko and Godin, 1990). In a group of infants with severe HIE, the i.v. administration of 40 mg/kg of allopurinol decreased the formation of free radicals (Van et al., 1998). In line with these results, administration of allopurinol i.v. to the mother during the birth of fetuses with hypoxia or incipient hypoxia has been shown to reduce the blood levels of S-100 protein in the umbilical artery (a biomarker of cerebral damage) (Torrance et al., 2009). A clinical trial is currently underway (Effect of Allopurinol for Hypoxic-ischemic Brain Injury on Neurocognitive Outcome, NCT03162653), to assess the therapeutic potential of allopurinol administered in the first minutes of life.

Interest in the use of stem cells to treat all kinds of diseases, among them HIE, is increasing (Nitkin et al., 2019). This therapy could facilitate repair and regeneration of damaged brain tissue after HI aggression via its interaction with immune system cells located in distant organs of the brain (e.g., spleen), thereby altering the immune/inflammatory response, as well as by interaction between the transplanted cells and the brain tissue to increase cell proliferation and neurogenesis (Bennet et al., 2012). Stem cell therapy of HIE, exclusive or associated with hypothermia, is still requires clinical trials to determine, among others, the most effective type of cells, the optimal dose and the most appropriate administration period for obtaining the best therapeutic results. One of these works in progress in the recruitment phase (Study of hCT-MSC in Newborn Infants With Moderate or Severe HIE, NCT03635450) will include a sample of 6 infants of 36 or more weeks of gestation with moderate-severe HIE, treated with hypothermia and infusion of two doses of stromal mesenchymal cells derived from umbilical cord.

Noble gases such as xenon and argon displayed neuroprotective activity in experimental models of HIE (Lobo et al., 2013). Their effect is mediated via their ability to decrease excitotoxicity by modulating glutamatergic NMDA receptors (Ma et al., 2007). The Total Body multicenter clinical trial hypothermia plus Xenon (TOBY-Xe) employed this gas plus hypothermia in 92 infants from 36 and 43 weeks (Azzopardi et al., 2016). To further examine some of the variables that may have influenced the treatment with this gas (i.e., dose or duration of treatment), a phase II clinical trial called CoolXenon3 Study (NCT02071394) is presently undergone.

Melatonin has been proposed as a promising strategy for HIE (Paprocka et al., 2019). A key point for melatonin efficacy to be used as therapy in HIE lies in its remarkable antioxidant and anti-inflammatory effects and in its capacity to cross readily the blood brain barrier (Hardeland et al., 2015; Reiter et al., 2017). Melatonin increased the level of protection developed by hypothermia by improving brain energy metabolism in a study employing piglets (Robertson et al., 2013). Clinically, treatment of asphyctic newborns with hypothermia and melatonin orally, reduces serum levels of oxidants more efficiently than hypothermia alone (Aly et al., 2015) and improved survival (Ahmad et al., 2018).

## Basic Biology of Melatonin Relevant to HIE

Circulating melatonin is produced primarily by the pineal gland at night (Claustrat and Leston, 2015). It provides circadian and seasonal timing cues of the length of the night by acting as a chronobiotic (Pandi-Perumal et al., 2008). In addition, almost every cell in the body having mitochondria produces melatonin and the intracellular concentrations of melatonin are much higher than those circulating in blood (Venegas et al., 2012; Reiter et al., 2017; Suofu et al., 2017). Intracellular melatonin does not get the extracellular space and doses of melatonin much higher than those employed as a chronobiotic are needed to modify its intracellular levels (Cardinali, 2019a).

MT_1_ and MT_2_ melatonin receptors belong to the superfamily of membrane receptors associated with G proteins (G-protein coupled receptors, GPCR) (Dubocovich et al., 2010). Another GPCR member, GPR50, was recently added to the melatonin receptor subfamily displaying high sequence homology with MT_1_ and MT_2_ but showing null binding capacity to melatonin. Homo- and heteromers among each other and also with other GPCRs are formed (Cecon et al., 2017).

Neuroprotection by melatonin is mediated via receptor and non-receptor mechanisms like antioxidant defense, improvement of energy metabolism and immune function, as well as anti-inflammatory, antiapoptotic and antiexcitotoxic effects (for ref. see Cardinali, 2019b).

Melatonin scavenges directly ROS (Hardeland et al., 1993; Reiter et al., 2000) and is further metabolized into strong antioxidant molecules (Galano et al., 2013; Tan et al., 2015). It also induces the upregulation of antioxidant enzymes like glutathione peroxidase, glutathione reductase, and superoxide dismutase (Fischer et al., 2013; Reiter et al., 2017). Melatonin decreases the release of pro-apoptotic proteins in response to injury and prevents apoptosis via stabilization of mitochondrial function (Tan and Reiter, 2019).

In rodent immature brain HI drives cell death via apoptosis through Bcl-2 family members (Morciano et al., 2016). As a consequence, mitochondria permeabilize, and proapoptotic factors, such as cytochrome C and the apoptosis-inducing factor are released into the cytoplasm. By increasing Bcl-2 protein expression and blocking Bax proapoptotic activity via the sirtuin (SIRT)-1/nuclear factor κB (NF-κB) axis, melatonin inhibits significantly cytochrome C release and caspase 3 activation (Sun et al., 2002; Tan and Reiter, 2019).

Mitochondria permeabilize due to the opening of mitochondrial permeability transition pore (mPTP). This is a pathophysiological event that leads to mitochondrial depolarization, swelling, and the activation of the apoptotic and necrotic pathways (Morciano et al., 2017). Melatonin protects from mitochondrial swelling and membrane depolarization (Waseem et al., 2016) and prevents cytochrome C release and cardiolipin peroxidation (Petrosillo et al., 2009) in isolated rodent brain mitochondria subjected to Ca^2+^-induced mPTP.

Melatonin is also an immunological modulator that shows remarkable anti-inflammatory properties (Carrillo-Vico et al., 2013; Hardeland, 2018). Because these properties are observed in high-grade inflammation such as sepsis, ischemia/reperfusion and brain injury, and in the low-grade inflammation seen in neurodegenerative disorders and aging, the anti-inflammatory actions are of great medical interest. Melatonin inhibits the binding of NF-κB to DNA, thus decreasing the synthesis of proinflammatory signals (Carrillo-Vico et al., 2013). It also inhibits cyclooxygenase (Cox) (Cardinali et al., 1980), particularly Cox2 (Deng et al., 2006), and decreases mRNA of inducible nitric oxide synthase (Costantino et al., 1998). Among the several signaling pathways involved in the anti-inflammatory action of melatonin (Hardeland, 2018), the upregulation of SIRT-1, which shares various effects known from melatonin and further interferes with the proinflammatory signaling, is considered of major importance (Tan and Reiter, 2019). Ultimately, these effects of melatonin lead to down-regulation of proinflammatory and up-regulation of anti–inflammatory cytokines (Hardeland, 2018).

Anti-excitotoxic actions also arise after melatonin administration. Relevant to this, melatonin curtails neuronal death induced by the ionotropic glutamate receptor agonist kainate (Giusti et al., 1996). In addition, melatonin administration reduces the injury of hippocampal CA1 neurons brought about by ischemia (Cho et al., 1997) or by high doses of glucocorticoids (Furio et al., 2008). Melatonin anti-excitotoxic activity does not involve melatonin receptors (Escames et al., 2004).

Another neuroprotective mode of action involves the γ-aminobutyric acid (GABA)-ergic system. Melatonin has anti-excitatory, and at enough dosage, sedating effects via GABAergic mechanisms (Golombek et al., 1996; Caumo et al., 2009) exerted via allosteric modulation of melatonin of GABA_A_ receptors (Cheng et al., 2012).

As far as a potential therapy for HIE in newborns, melatonin has many advantages. It crosses readily the blood-brain barrier, its antioxidant and anti-inflammatory effects are readily exerted and it has an excellent safety profile (Gitto et al., 2001, 2009; Welin et al., 2007; Foley and Steel, 2019).

## Melatonin in Experimental Models of HIE

Table 1 summarizes information on melatonin activity in animal models of HIE. With a few exceptions (Berger et al., 2016), a compelling amount of evidence supports the efficacy of melatonin on long-term consequences of a neonatal HI brain injury in rats and mice (behavioral asymmetry, learning deficits, etc.) (Jantzie et al., 2018).

**TABLE 1:** Melatonin activity in animal models of HIE.

**Findings**	**Melatonin dose**	**References**
Melatonin provides neuroprotection in the late-gestation fetal sheep brain in response to umbilical cord occlusion	1 mg bolus i.v., then 1 mg/h for 2 h	Miller et al. (2005)
Melatonin protects from the long-term consequences of a neonatal hypoxic-ischemic brain injury in rats (behavioral asymmetry, learning deficits)	15 mg/kg i.p.	Carloni et al. (2008)
In 1-day-old Wistar rats subjected to hypoxia melatonin treatment reduced VEGF and NO levels as well as leakage of horseradish peroxidase in choroid plexus	10 mg/kg i.p.	Sivakumar et al. (2008)
Melatonin normalizes free iron, total isoprostanes, and total neuroprostanes in a rat model of neonatal HI encephalopathy	15 mg/kg i.p.	Signorini et al. (2009)
Melatonin was not able to reduce cortical infarct volume in a rat neonatal stroke model but strongly reduces inflammation and promotes subsequent myelination in the white matter	20 mg/kg i.p. (two doses)	Villapol et al. (2011)
In neonatal rats subjected to HI melatonin reduced the percent infarcted brain volume and TUNEL positivity	20 mg/kg i.p.	Cetinkaya et al. (2011)
Treatment with melatonin after neonatal HI in rats led to a neuroprotective effect reducing cell death, white matter demyelination and reactive astrogliosis	15 mg/kg i.p.	Alonso-Alconada et al. (2012)
Melatonin reduces oxidative stress and inflammatory cells recruitment and glial cells activation in cerebral cortex after neonatal HI damage of rats	15 mg/kg i.p.	Balduini et al. (2012)
In a neonatal rat model of HI brain injury, melatonin, and topiramate, administered either alone or in combination significantly reduced the percent infarcted brain volume and number of TUNEL positive cells	20 mg/kg i.p.	Ozyener et al. (2012)
In a piglet model of perinatal asphyxia, melatonin-augmented hypothermia significantly reduced the hypoxic-ischemic-induced increase of lactate/*N*-acetyl aspartate and lactate/total creatine ratios in the deep gray matter. Melatonin-augmented hypothermia increased levels of brain nucleotide triphosphate/exchangeable phosphate pool. Correlating with improved cerebral energy metabolism, TUNEL-positive nuclei were reduced in the hypothermia plus melatonin group compared with hypothermia alone in the thalamus, internal capsule, putamen and caudate, and there was reduced cleaved caspase 3 in the thalamus	5 mg/kg/h over 6 h started at 10 min after resuscitation and repeated at 24 h	Robertson et al. (2013)
In neonatal rats subjected to HI, melatonin administration reduced the neuron splicing of XBP-1 mRNA, the increased phosphorylation of eIF2α, and elevated expression of chaperone proteins GRP78 and Hsp70. Melatonin also prevented the depletion of SIRT-1 induced by HI	15 mg/kg i.p.	Carloni et al. (2014)
Melatonin prevents cell death and mitochondrial dysfunction via a SIRT1-dependent mechanism during ischemic-stroke in mice	10 mg/kg twice	Yang et al. (2015)
No improvement of neuronal metabolism in the reperfusion phase with melatonin treatment after HI brain injury in the neonatal rat was seen	10 mg/kg i.p.	Berger et al. (2016)
In a neonatal rat model of HI brain injury, the integrity of the auditory pathway in the brainstem was preserved by melatonin treatment	15 mg/kg i.p.	Revuelta et al. (2016)
In a rat neonatal model of HIE, melatonin reduced necrotic cell death and decreased activation of the early phases of intrinsic apoptosis, with a concomitant increased expression and activity of SIRT1, reduced expression and acetylation of p53, and increased autophagy activation	15 mg/kg i.p.	Carloni et al. (2017b)
Melatonin alleviates brain and peripheral tissue edema in a neonatal rat model of HIE, as assessed by expression of the edema related proteins AQP-4, ZO-1, and occludin	10 mg/kg i.p.	Xu et al. (2017)
In postnatal day 7 rat pups subjected to unilateral HI, pre-treatment with melatonin significantly reduced brain damage with 30% recovery in tissue loss compared to vehicle-treated animals. Autophagy and apoptotic cell death were significantly inhibited after melatonin treatment	15 mg/kg i.p.	Hu et al. (2017)
Characterization of gene expression in the rat brainstem after neonatal HI injury melatonin has retarded effects on gene activation	15 mg/kg i.p.	Revuelta et al. (2017)
After acute HI insult in preterm fetal sheep, melatonin administration decreased apoptosis, inflammation and oxidative stress within the white matter. It also increased oligodendrocyte cell number within the periventricular white matter only and improved myelin density within the subcortical but not the striatal white matter	0.2 mg bolus i.v. to the fetus at 2 h after HI followed by an infusion of 0.1 mg/h for 24 h	Yawno et al. (2017)
Melatonin acts in synergy with hypothermia to reduce oxygen-glucose deprivation-induced cell death in rat hippocampal slices	25 μM	Carloni et al. (2018)
Melatonin protects from newborn hypoxic-ischemic brain injury melatonin in murine experimental models through MT_1_ receptor	5–10 mg/kg i.p.	Sinha et al. (2018)
Repetitive neonatal melatonin treatment prevents from functional deficits in a rat model of cerebral palsy	20 mg/kg i.p.	Jantzie et al. (2018)
In a lamb model of perinatal asphyxia melatonin (i.v. or as a transdermal patch) alleviated acidosis and altered determinants of encephalopathy. Asphyxia significantly increased brain white and gray matter apoptotic cell death, lipid peroxidation and neuroinflammation, all effects mitigated by melatonin	60 mg in 24 h; i.v. or transdermal patch	Aridas et al. (2018)
Melatonin was administered at 2 h and 6 h after hypoxia-ischemia with cooling in a piglet model. Neuroprotection was dose dependent; 15 mg/kg melatonin started 2 h after HI, given over 6 h, was well tolerated and augmented hypothermic protection in sensorimotor cortex	5 or 15 mg/kg i.v.	Robertson et al. (2019)

*VEGF, vascular endothelial growth factor; NO, nitric oxide; AQP-4, aquaporin 4.*

Treatment with melatonin after neonatal HI in rats led to a neuroprotective effect reducing cell death, white matter demyelination and reactive astrogliosis (Alonso-Alconada et al., 2012; Hu et al., 2017). Melatonin prevents cell death and mitochondrial dysfunction via a SIRT1-dependent mechanism during ischemic-stroke (Yang et al., 2015; Carloni et al., 2017b). Melatonin reduced necrotic cell death and decreased activation of the early phases of intrinsic apoptosis, with a concomitant increased expression and activity of SIRT1, reduced expression and acetylation of p53 and increased autophagy activation (Xu et al., 2017).

The effect of melatonin was also apparent in other models of HIE. In a piglet model of perinatal asphyxia, melatonin-augmented hypothermia reduced the HI-induced increase of lactate/*N*-acetyl aspartate and lactate/total creatine ratios in the deep gray matter. Apoptosis was reduced in the hypothermia plus melatonin group in the thalamus, internal capsule, putamen and caudate, and there was reduced cleaved caspase 3 in the thalamus (Robertson et al., 2013). In the late-gestation fetal sheep brain in response to umbilical cord occlusion melatonin provided neuroprotection by decreasing lipid peroxidation (Miller et al., 2005). In another study with acute HI insult in preterm fetal sheep, melatonin administration decreased apoptosis, inflammation and oxidative stress within the white matter (Yawno et al., 2017).

## Melatonin in HIE: Clinical Studies

Table 2 summarizes melatonin-related clinical observations in HIE.

**TABLE 2:** Studies including treatment of HIE patients with melatonin.

**Subjects**	**Design**	**Melatonin dose**	**Measured**	**Results**	**References**
20 asphyxiated newborns	Open-label study	10 HIE newborns were treated with a total of 80 mg as eight oral doses	Serum malondi-aldehyde and nitrite/nitrate concentration	In the asphyxiated newborns given melatonin, there were significant reductions in malondialdehyde and nitrite/nitrate levels at both 12 and 24 h. Three of the 10 asphyxiated children not given melatonin died within 72 h after birth; none of the 10 asphyxiated newborns given melatonin died	Robertson et al. (2019)
74 preterm infants with respiratory distress syndrome	Open-label study	40 preterm infants were treated with a total of 100 mg/kg as 10 infusions	IL-6, IL-8, TNFα in tracheobronchial aspirate and serum nitrite/nitrate concentration	Compared with the melatonin-treated respiratory distress syndrome newborns, in the untreated infants the concentrations of IL-6, IL-8, and TNFα 7 days after onset of the study were higher. In addition, nitrite/nitrate levels at all time points were higher in the untreated respiratory distress syndrome newborns than in the melatonin-treated babies	Fulia et al. (2001)
120 preterm infants with respiratory distress syndrome	Open-label study	60 preterm infants were treated with a total of 100 mg/kg as 10 infusions	Serum IL-6, IL-8, TNFα and nitrite/nitrate concentration	Melatonin treatment reduced the proinflammatory cytokines and improved the clinical outcome	Gitto et al. (2004b)
110 preterm infants with respiratory distress syndrome	Open-label study	55 HIE newborns were treated with a total of 100 mg/kg as 10 infusions	IL-6, IL-8, TNFα in trachea-bronchial aspirate and serum nitrite/nitrate concentration	Melatonin treatment reduced the proinflammatory cytokines and improved the clinical outcome	Gitto et al. (2004a)
18 preterm infants	Open-label study	Total of 0.04–0.6 μg/kg over 0.5–6 h as an infusion	Pharmaco-kinetic profiles	The pharmacokinetic profile of melatonin in preterm infants differs from that of adults so dosage of melatonin for preterm infants cannot be extrapolated from adult studies	Gitto et al. (2005)
30 HIE newborns, 15 healthy newborns	Randomized prospective trial	15 HIE newborns were treated with a total of 50 mg/kg as five daily enteral doses	Serum melatonin, plasma SOD, serum NO, EEG, MRI, neurologic evaluations	At day 5, the melatonin/hypothermia group had greater increase in melatonin and decline in NO and less decline in SOD. The melatonin/hypothermia group had fewer seizures on follow-up EEG and less white matter abnormalities on MRI. At 6 months, the melatonin/hypothermia group had improved survival without neurological or developmental abnormalities	Aly et al. (2015)
15 preterm infants; 5 preterm infants with low dose, 5 preterm infants with medium dose, 5 preterm infants with high dose	Open-label study	Total of 0.5 mg/kg or 3 mg/kg or 15 mg/kg as 1 or 3 intragastric boluses	Pharmacokinetic profiles	A different pharmacokinetic profile in premature newborns, compared to adults. The high peak plasma concentrations and the long half-life indicate that in the neonatal clinical setting, it is possible to obtain and maintain high serum concentrations using a single administration of melatonin repeated every 12/24 h	Merchant et al. (2013)
80 HIE newborns	Randomized prospective trial	40 HIE newborns received melatonin 10 mg orally via nasogastric tube at admission	Newborns were followed for 28 days to see the effect of melatonin in terms of survival rate	Administration of melatonin as an adjunct therapy in the management of newborns with HIE led to improved survival rate	Ahmad et al. (2018)
5 neonates with HIE undergoing hypo-thermia	Open-label study	Melatonin was infused at 0.5 mg/kg	Pharmacokinetic profiles	Melatonin half-life and clearance were prolonged, and the distribution volume decreased compared to adults. Hypothermia did not affect melatonin pharmacokinetics	Carloni et al. (2017a)

*IL, interleukin; TNF, tumor necrosis factor; SOD, superoxide dismutase; NO, nitric oxide.*

An initial observation indicated that in asphyxiated newborns with HIE, oral administration of melatonin (80 mg in eight doses) reduced serum malondialdehyde and nitrite/nitrate concentrations and improved survival (Fulia et al., 2001). Subsequent reports from the same group of investigators indicated that in preterm infants with respiratory distress the treatment with 100 mg/kg in 10 infusions improved the clinical outcome and reduced IL-6, IL-8, and TNFα concentrations in tracheobronchial aspirate and serum nitrite/nitrate concentration (Fulia et al., 2001; Gitto et al., 2004a, b, 2005; Hu et al., 2017; Revuelta et al., 2017; Yawno et al., 2017; Aridas et al., 2018; Carloni et al., 2018; Sinha et al., 2018; Robertson et al., 2019).

In a randomized prospective trial including 30 HIE newborns treatment with 50 mg/kg of melatonin as five daily enteral doses the melatonin/hypothermia group had greater increase in melatonin and decline in circulating oxidants, fewer seizures in EEG and less white matter abnormalities in magnetic resonance imaging. At 6 months, the melatonin/hypothermia group had improved survival without neurological or developmental abnormalities (Aly et al., 2015). A similar improvement of survival was reported in another randomized prospective trial including 40 HIE newborns receiving melatonin 10 mg orally via nasogastric tube (Ahmad et al., 2018).

Concerning the melatonin doses employed, it must be noted that the pharmacokinetic profile of melatonin in preterm infants differs from that of adults, making it impossible to applied allometric calculations for establishment of the optimal doses derived from studies in adults (Merchant et al., 2013). The high peak plasma concentrations and the long half-life of melatonin in newborn indicate that in the neonatal clinical setting, it is possible to obtain and maintain high serum concentrations of melatonin using a single administration repeated every 12/24 h (Carloni et al., 2017a). Moreover, hypothermia does not affect melatonin pharmacokinetics (Balduini et al., 2019).

## Concluding Remarks

A remarkable number of melatonin effects strongly suggest that it may have an important role therapeutic role in HIE. Melatonin has antiexcitotoxic, anti-apoptotic, anti-inflammatory and antioxidant effects in a number of animal models of HIE and modulates normal glial development (Table 1). Clinically, randomized controlled pilot trials evaluating melatonin as an adjuvant to hypothermia in HIE indicated that the melatonin/hypothermia group show a reduced number of seizures, less evidence of white matter injury and a lower rate of mortality without developmental or neurological abnormalities (Aly et al., 2015; Ahmad et al., 2018).

Melatonin is remarkably non-toxic, and its safety is very high. The lethal dose 50 for the i.p. injection of melatonin was determined for rats and mice (1168 and 1131 mg/kg), but failed to be measured after the oral administration of up to 3200 mg/kg to rats or of the s.c. injection of up to 1600 mg/kg to rats and mice (Sugden, 1983).

Melatonin shows a high safety profile in humans (Cardinali, 2019b; Foley and Steel, 2019) and, in general, is very well tolerated. Therefore, melatonin holds a promise in management of infants with HIE (Hendaus et al., 2016). Currently, the MELPRO study (NCT03806816) is in the process of recruiting patients, with the aim to include 100 newborns. This and additional phase III clinical trials are essential for the subsequent application of melatonin in newborn with HIE. Unfortunately, the pharmaceutical industry is refractive to support studies on melatonin because of the lack of protective patents for a natural compound. Hence, only with the involvement of governmental and non-profit organizations such a goal can be achieved.

## Author Contributions

The author confirms being the sole contributor of this work and has approved it for publication.

## Conflict of Interest

The author declares that the research was conducted in the absence of any commercial or financial relationships that could be construed as a potential conflict of interest.
